# Guest-Host Chemistry with Dendrimers—Binding of Carboxylates in Aqueous Solution

**DOI:** 10.1371/journal.pone.0138706

**Published:** 2015-10-08

**Authors:** Mario Ficker, Johannes F. Petersen, Jon S. Hansen, Jørn B. Christensen

**Affiliations:** 1 Department of Chemistry, University of Copenhagen, Thorvaldsensvej 40, DK- 1871 Frederiksberg C, Denmark; Brandeis University, UNITED STATES

## Abstract

Recognition and binding of anions in water is difficult due to the ability of water molecules to form strong hydrogen bonds and to solvate the anions. The complexation of two different carboxylates with 1-(4-carbomethoxypyrrolidone)-terminated PAMAM dendrimers was studied in aqueous solution using NMR and ITC binding models. Sodium 2-naphthoate and sodium 3-hydroxy-2-naphthoate were chosen as carboxylate model compounds, since they carry structural similarities to many non-steroidal anti-inflammatory drugs and they possess only a limited number of functional groups, making them ideal to study the carboxylate-dendrimer interaction selectively. The binding stoichiometry for 3-hydroxy-2-naphthoate was found to be two strongly bound guest molecules per dendrimer and an additional 40 molecules with weak binding affinity. The NOESY NMR showed a clear binding correlation of sodium 3-hydroxy-2-naphthoate with the lyophilic dendrimer core, possibly with the two high affinity guest molecules. In comparison, sodium 2-naphthoate showed a weaker binding strength and had a stoichiometry of two guests per dendrimer with no additional weakly bound guests. This stronger dendrimer interaction with sodium 3-hydroxy-2-naphthoate is possibly a result of the additional interactions of the dendrimer with the extra hydroxyl group and an internal stabilization of the negative charge due to the hydroxyl group. These findings illustrate the potential of the G4 1-(4-carbomethoxy) pyrrolidone dendrimer to complex carboxylate guests in water and act as a possible carrier of such molecules.

## Introduction

Dendrimers are well-defined nano-scale macromolecules formed by repetitive branching from a core. Depending on the branch-cell unit, dendrimers can have cavities capable of hosting smaller molecules. Guest-host chemistry in dendrimers is divided into endo- or exo-complexation which is determined by whether the guest molecule is bound in the interior or to the surface of the dendrimer. Both types of guest-host chemistry have been a popular topic due to the potential applications in drug-delivery.[1–4] 1-(4-Carbomethoxy) pyrrolidone coated PAMAM dendrimers are especially promising candidates for the complexation and release of drug molecules, since they have unique and favorable solubility properties in both organic solvents and aqueous solutions[5] and have a benign toxicity profile.[6–8]

We recently reported a study of endo-complexation of the γ-lactam antibiotic oxacillin in a G4 1,4-diaminobutane-core 1-(4-carbomethoxy) pyrrolidone functionalized PAMAM-dendrimer, where it was found that the stoichiometry of the guest-host complexes showed solvent dependency.[9]

However, oxacillin and the other penicillins are sold as alkali metal salts due to the low stability of the free carboxylic acids; this raised the question of whether it could be possible to have binding of carboxylate anions to the pyrrolidone-terminated dendrimer in water.

Recognition and binding of anions in water is difficult because of waters ability to form strong hydrogen bonds and to solvate the anions. Many of the best examples of anion receptors are pre-organized macromolecules with suitable cavities such as cryptands,[10] calixarenes,[11] or curcubiturils.[12] Guest-host chemistry with dendrimers in water is much less investigated, but there are examples of binding of pharmaceutically interesting compounds such as cis-Platin,[13] Nadifloxacine[14] and Prulifloxacine,[15] Campthotecin,[16] Dexamethasone phosphate,[17] anti-inflammatoric drugs (NSAIDs)[18,19] and of course DNA and siRNA.[20–23]

**Fig 1:**
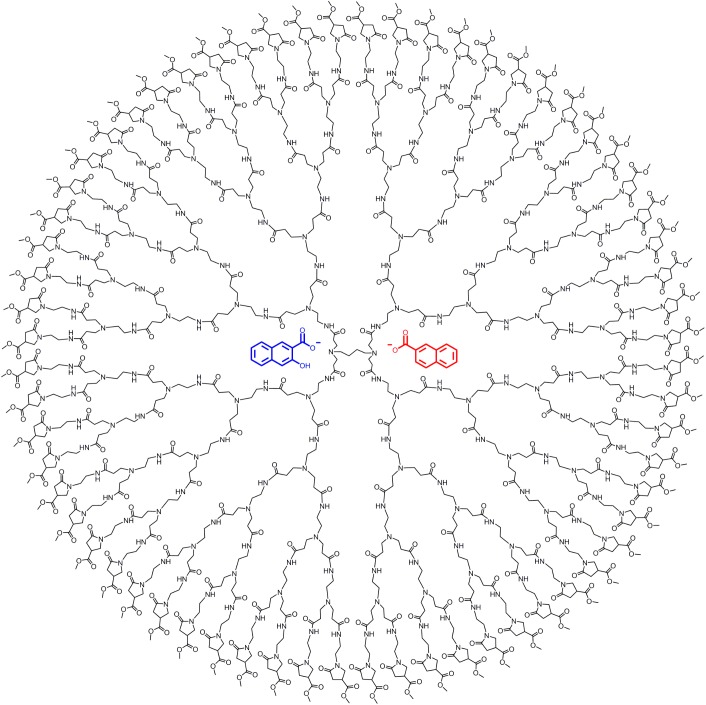
The two model guests illustrated within a G4 1-(4-carbomethoxy) pyrrolidone dendrimer.

Initially, we tried the sodium salt of oxacillin, but because the results were inconclusive, we decided to look at the more simple molecules such as sodium 2-naphthoate and sodium 3-hydroxy-2-napthoate. These two carboxylates were chosen as model guests, since they possess similar structural features and water solubility as many antibiotics and non-steroidal anti-inflammatory drugs. The G4 1-(4-carbomethoxy) pyrrolidone dendrimer and the two encapsulated guest molecules are illustrated in Fig 1.

## Materials and Methods

Unless otherwise stated, all starting materials were obtained from commercial suppliers and used as received. Solvents were HPLC grade and used as received. ^1^H-NMR spectra were recorded on a 500 MHz NMR (Bruker) apparatus. Chemical shifts are reported in ppm downfield of TMS (tetramethylsilane) using the resonance of the deuterated solvent as internal standard (s = singlet, d = doublet, t = triplet, q = quartet, m = multiplet). ^1^H-NMR titration data were fitted with Origin 9.0. The employed ITC apparatus was a NanoITC Model 5300, TA Instruments, Lindon, UT, USA, with a cell volume of 1038 μl. All ITC data were fitted in NanoAnalyze.

### Preparation of dendrimers

The PAMAM dendrimer of the generation 4 was synthesized by published procedures,[24] starting from 1,4-diaminobutane as the core. The 1-(4-carbomethoxy-pyrrolidone) surface functionalization was done by reacting the amino terminated dendrimers with dimethyl itaconate (S1 Fig and S1 Text).[6,25] The reaction was monitored by performing Kaiser-tests until completion.

### Preparation of the corresponding sodium salt of the naphthoic acids

Sodium 3-hydroxy-2-naphthoate and sodium 2-naphthoate were prepared by the slow addition of the corresponding carboxylic acid (2.9 mmol) to an aqueous solution (10 mL) containing an equivalent amount of NaOH (2.9 mmol). Excess water was removed by freeze drying and the carboxylates were gained in a quantitative yield (2.9 mmol).

Sodium 3-hydroxy-2-naphthoate: ^1^H-NMR: δ = 8.33 (s, 1H); 7.85–7.88 (m, 1H); 7.69–7.72 (m, 1H); 7.50–7.53 (m, 1H); 7.35–7.38 (m, 1H); 7.18 (s, 1H). Sodium 2-naphthoate: ^1^H-NMR: δ = 7.97–8.02 (m, 1H); 7.74–7.80 (m, 2 H); 7.32–7.44 (m, 4 H).

### Preparation of NMR-Titration samples and dissociation constant determination

5 ml stock solutions in D_2_O were prepared, containing 1 mM G4 1-(4-carbomethoxy pyrrolidone) dendrimer and (**2**) containing 1 mM of G4 1-(4-carbomethoxy pyrrolidone) dendrimer and 150 mM of the respective carboxylate. The dendrimer concentration was kept constant, while the carboxylate concentration was varied from 0 to 100 mM. For the determination of the dissociation constant of sodium 2-naphthoate, a NMR fitting model was used as described in an earlier paper.^4^ Binding saturation curves were fitted employing *Origin 9*.*0*.

### Preparation of samples for 2D-NOESY-experiments

NOESY experiments were conducted on a 500 MHz NMR (Bruker) apparatus that was equipped with a cryo-probe. The concentration of G4 1-(4-carbomethoxypyrrolidone) PAMAM-dendrimer was 5.2 mM and the concentration of sodium 3-hydroxy-2-naphthoate was 35.7 mM in D_2_O. The dendrimer sample was incubated at room temperature with sodium 3-hydroxy-2-naphthoate for 2 hours prior to starting the experiment. The concentration of sodium 2-naphthoate incubated with dendrimer in D_2_O was the same. The experiments were performed at 25°C with a 2 s relaxation delay, 205 ms acquisition time, 300 ms mixing time and a 8.2 μs ^1^H 90° pulse width. Eight transients were averaged for 1024 *t1* increments.

### Preparation of NMR-samples for Job-plot

5 ml stock solutions in D_2_O were prepared with 10 mM of G4 1-(4-carbomethoxy pyrrolidone) dendrimer, and 10 mM of the respective carboxylate. The samples were prepared by injecting a total volume of 500 μl in 12 NMR tubes for each Job-plot, keeping a constant total concentration of 10 mM ([*den*] + [*carboxylate*]), where the ratio [*den*]/[*carboxylate*] was varied.

#### ITC-titration experiments

ITC measurements have been performed following an ITC best practice guideline by Freyer and Lewis [26]. For each compound there have been 42 individual heat signals collected. The computational fitting of the data was performed using the *NanoAnalyze* standard software for ITC measurements by TA Instruments (manufacturing company of the NanoITC Model 5300). Before and after the measurements the accuracy of the instrument was tested by standardized blank titration of water into water. Control experiments for baseline determination were performed (blank titration of the carboxylate into water and a blank titration of water into the dendrimer solution). The error values of the obtained values (fitting by the *NanoAnalyze* standard instrument software) are reported after each value.

### Titration of G4 4-carbomethoxy pyrrolidone terminated PAMAM-dendrimer with sodium 3-hydroxy-2-naphthoate

A stock solution of 0.1 mM G4 4-carbomethoxy pyrrolidone terminated PAMAM-dendrimer in MQ water was prepared (2 ml), **A**. 40 mM sodium 3-hydroxy-2-naphthoate was prepared (5 ml), **B**. Both solutions **A** and **B** were carefully degassed by ultra sonification in order to remove the air content. The pH of the aqueous carboxylate solutions was adjusted to match the pH of the 0.1 mM dendrimer solution (pH 7.8). The ITC cell was filled with solution **A** (1038 μl). The ITC syringe (250 μl) was filled with solution **B**. The temperature was maintained at 25°C throughout the experiment. Addition of solution **B** into solution **A** was carried out by injection of 6 μl for each titration increment, giving rise to 42 heat signals.

### Baseline Subtraction Blank ITC-titration experiments

The ITC cell was filled with solution **A** (1038 μl). The ITC syringe (250 μl) was filled with MQ water, which was carefully degassed by ultra sonification. Addition of MQ water into solution **A** was carried out by injection of 6 μl for each titration increment, giving rise to 42 heat signals.

Blank titration of solution **B** into MQ water was performed by filling the ITC cell with 1038 μl of degassed MQ water followed by the addition of 250 μl of solution **B**. 6 μl of solution **B** was added for each titration increment, giving rise to 42 heat signals. The same blank titration experiment was conducted with sodium 2-naphthoate as described for sodium 3-hydroxy-2-naphthoate. Another blank experiment was conducted by addition of water to the dendrimer solution. This did not result in a significant heat signal and was thus neglected.

## Results and Discussion

The complexation of the two carboxylate compounds was studied by means of ^1^H-NMR- and ITC-titrations, these two techniques have been applied previously in similar studies.[27–30] Both analytical techniques showed guest encapsulation in aqueous solution. The dissociation constants were calculated from the obtained ITC data and a recently used ^1^H-NMR binding model was also applied to study the binding strength.[9]

**Fig 2:**
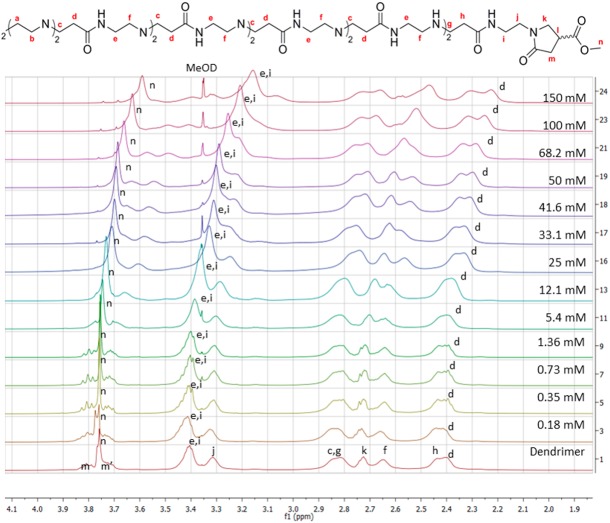
Stacked ^1^H-NMR spectra of different ratios of 3-hydroxy-2-naphthoate incubated with a 1 mM G4 1-(4-carbomethoxy) pyrrolidone dendrimer in D_2_O.

The complex formation of these carboxylates within the dendrimer was elucidated by a titration series of different concentrations of sodium 3-hydroxy-2-naphthoate and sodium 2-naphthoate incubated with a 1 mM aqueous solution of G4 1-(4-carbomethoxy) pyrrolidone dendrimer. The dendrimer signals underwent a significant change in chemical shift and line broadening as a consequence of guest encapsulation, as illustrated in Fig 2. The full titration series for both carboxylates, where the change in chemical shift for the host and guest molecules are shown, can be found in the supporting information (S4 and S5 Figs).

The inner dendrimer signals, **d** and **e**, experience a particularly large shift (0.1 ppm and 0.25 ppm respectively) due to the possible close proximity of the corresponding guest molecule. At increased guest concentration, the surface protons **m** and **n** also show a change in chemical shift. This binding mode is most likely caused by a combination of lipophilic and electrostatic interactions between the carboxylate and the dendrimer. The electrostatic interaction presumably occurs between the negatively charged carboxylate and the partially protonated interior of the dendrimer, i.e. the tertiary amine focal points.

**Fig 3:**
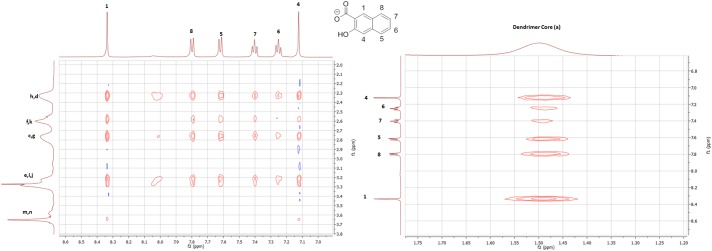
2D-NOE-spectrum showing a significant correlation between sodium 3-hydroxy-2-naphthoate and the G4 1-(4-carbomethoxypyrrolidone) PAMAM-dendrimer.

Job´s method was used to calculate the respective binding stoichiometries,[9] where sodium 2-naphthoate and sodium 3-hydroxy-2-naphthoate were both calculated to form 1:2 dendrimer-carboxylate ratios. The Job-plot experiments are shown in the supporting information (S6 Fig). It should be noted that the maxima calculated from the Job-plots only provide the stoichiometry that contributes most to the observed chemical shift. The found ratio is thus the predominant host-guest interaction, even though there might be different guest-host stoichiometries present in low concentrations. Two dimensional NOE experiments were conducted to obtain a deeper understanding of the binding phenomenon within the dendrimer cavity. The 3-hydroxy-2-naphthoate interacts with the core and interior branching of the dendrimer, as can be seen in Fig 3. In particular, the dendrimer proton signal **a** correlates with the aromatic guest protons, presumably due to a favorable lipophilic interaction with the butyl core. The dendrimer surface protons **m** and **n** did not give NOE correlations to the complexed carboxylate guests, this observation indicates that the binding motif is situated in the interior of the dendrimer. The full spectra for both carboxylates and a graphic illustration of the presumed binding site can be found in the supporting information (S7, S8 and S9 Figs).

**Fig 4:**
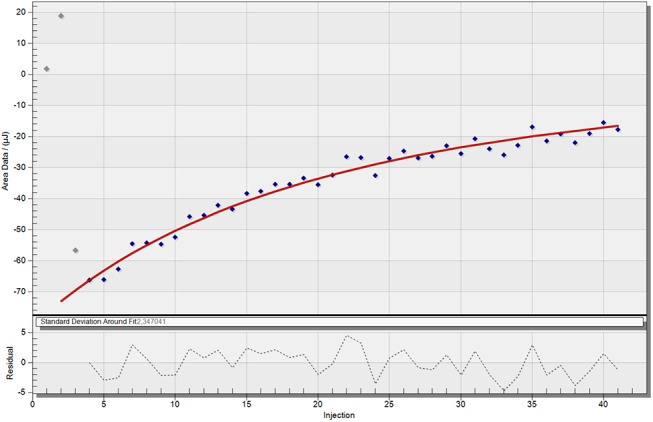
ITC-binding curve of 2-naphthoate, showing the best fit. The ITC raw data was treated by blank subtraction (titration of guest into water).

**Fig 5:**
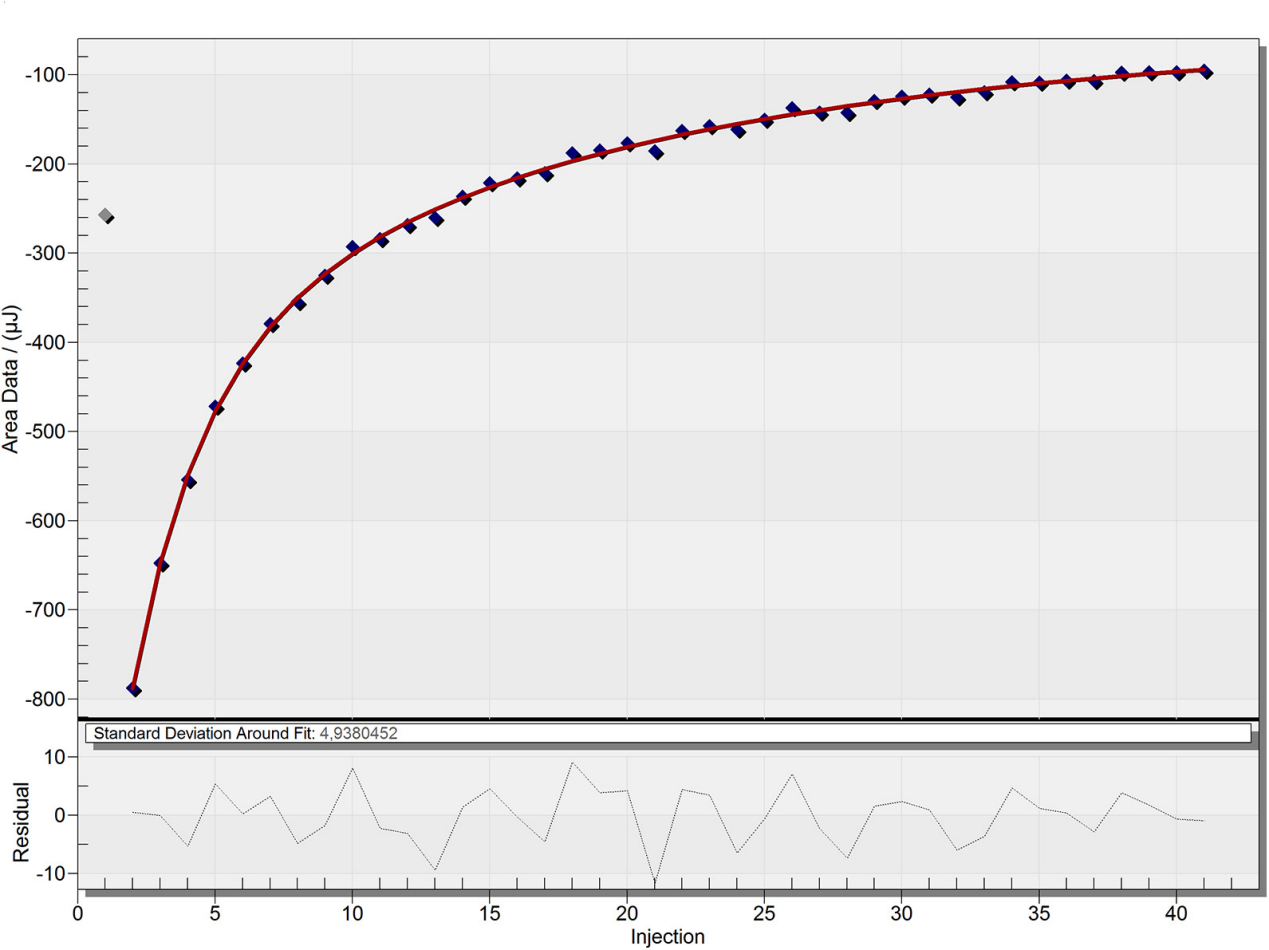
ITC-binding curve of 3-hydroxy-2-naphthoate, showing the best fit. The ITC raw data was treated by blank subtraction (titration of guest into water).

The association constants and binding enthalpies by complexation of the two carboxylates within the dendrimer were determined by ITC-experiments. Titration of a 40 mM solution of the respective carboxylate guest to a 0.1 mM solution of G4 1-(4-carbomethoxy) pyrrolidone dendrimer in water was performed in order to obtain the titration series (S10 and S11 Figs). A blank experiment for baseline subtraction was performed by gradual addition of the guest molecule solution into a dendrimer free aqueous solution (S12 and S13 Figs). Another blank experiment was conducted adding water to a dendrimer solution (S14 Fig). This did not result in a significant heat signal and was thus neglected in the determination methodology. The best fits for the binding curves are shown in Fig 4 and Fig 5.

The ITC-results (Table 1) show a difference in binding behavior of the two model guests. The sodium 2-naphthoate exhibits a weak binding interaction (K_*a*_ = 88.2 ± 8.4 M^-1^) with the dendrimer, showing a 2:1 stoichiometry. This ratio is in accordance with the determined ratio from the ^1^H-NMR Job-plot experiment. In comparison, the sodium 3-hydroxy-2-naphthoate exhibits a more complex binding interaction. The dendrimer shows a strong binding (K_*a1*_ *=* 2369 ± 927 M^-1^) with a ratio of 2:1 carboxylate to dendrimer. Again, this number of strong bound guest molecules is in accordance with the ^1^H-NMR determined stoichiometry. Additionally to this very strong binding interaction of two guest molecules, presumably at the lipophilic dendrimer core, an additional weak binding contribution was found during the ITC studies. Fitting of the heat signal indicated that two guest molecules alone were not the only contribution to the system; besides these two strongly bound guests, a larger number (app. 40) of guest molecules showed an additional low affinity binding (K_*a2*_ *=* 259 ± 101 M^-1^) to the dendrimer. This weak binding is visible in the long tailing of the heat signals over a broad range of high concentration ratios of guest to dendrimer. While the high affinity binding side is quickly occupied with 2 guest molecules, the weak binding takes places over a very broad range of concentration; and even at a dendrimer to guest ratio of 1:100, the dendrimer binding sites were not fully occupied, which indicates a very low binding affinity of the second guest molecule. Due to the low K_a2_ value, compared to the K_a1_, which is around 10 times stronger, it can be justified why the ^1^H-NMR Job plot only resulted in a 1:2 ratio of dendrimer to guest, since the Job-plot gives an out-read of the most dominant contribution to the system, which is in this case the strong K_a1_. This demonstrated, how important it is to apply multiple analytical techniques to get a full understanding of the binding behavior to a complex system like a dendrimer. Due to the more complex binding scenario in the case of the 3-hydroxy-2-naphthoate, the error bars for the association constants and enthalpies are larger compared to the 2-naphthoate. This is a consequence of the additional equation parameters, which are necessary when having an additional binding site and thus more variables that need to be fitted (see supporting information S1 Text and literature reference[26,31] for more details about the ITC equations).

**Table 1:** The association constants obtained from fitting of the ITC-data, along with calculated enthalpy increases.

	3-hydroxy-2-naphthoate	2-naphthoate
K_a1_ [M^-1^]	2369 ± 927	88.2 ± 8.4
K_a2_ [M^-1^]	259 ± 101	-
ΔH_1_ [kJ/mol]	-37 ± 8	-18.0 ± 1.7
ΔH_2_ [kJ/mol]	-87 ± 17	-
n_1_	2 ± 1	2.1 ± 0.2
n_2_	41 ± 24	-

The stronger binding of 3-hydroxy-2-naphthoate compared to 2-naphthoate may be explained by the higher lipophilicity of 3-hydroxy-2-naphthoate. The carboxylate unit in 3-hydroxy-2-naphthoate is capable of forming an intramolecular hydrogen bond with the adjacent hydroxyl group, resulting in a higher degree of negative charge delocalization and thus decreasing the overall hydrophilicity of the carboxylate. This intramolecular stabilization correlates well to the association of 3-hydroxy-2-naphthoate to the lipophilic interior of the dendrimer at low guest concentration, as illustrated by the conducted NOE experiments. At higher carboxylate concentration these preferred binding sites are already occupied, forcing additional carboxylate guests to associate with the more hydrophilic branches of the outer parts of the dendrimer.

Recently, we applied a binding model for ^1^H-NMR titrations in order to determine association constants between guest molecules and dendrimers.[9] In this binding model it is assumed that each dendrimer has a defined number of equal and independent binding sites in order for the algorithm to be applied to the system. For further information about this correlation of chemical shifts to the association constant we refer to this previously published study. Due to the requirement of equal binding sites this model could not be applied to the 3-hydroxy-2-naphthoate study, since the assumption of equal binding sites is not coherent with the previously discussed results.

**Fig 6:**
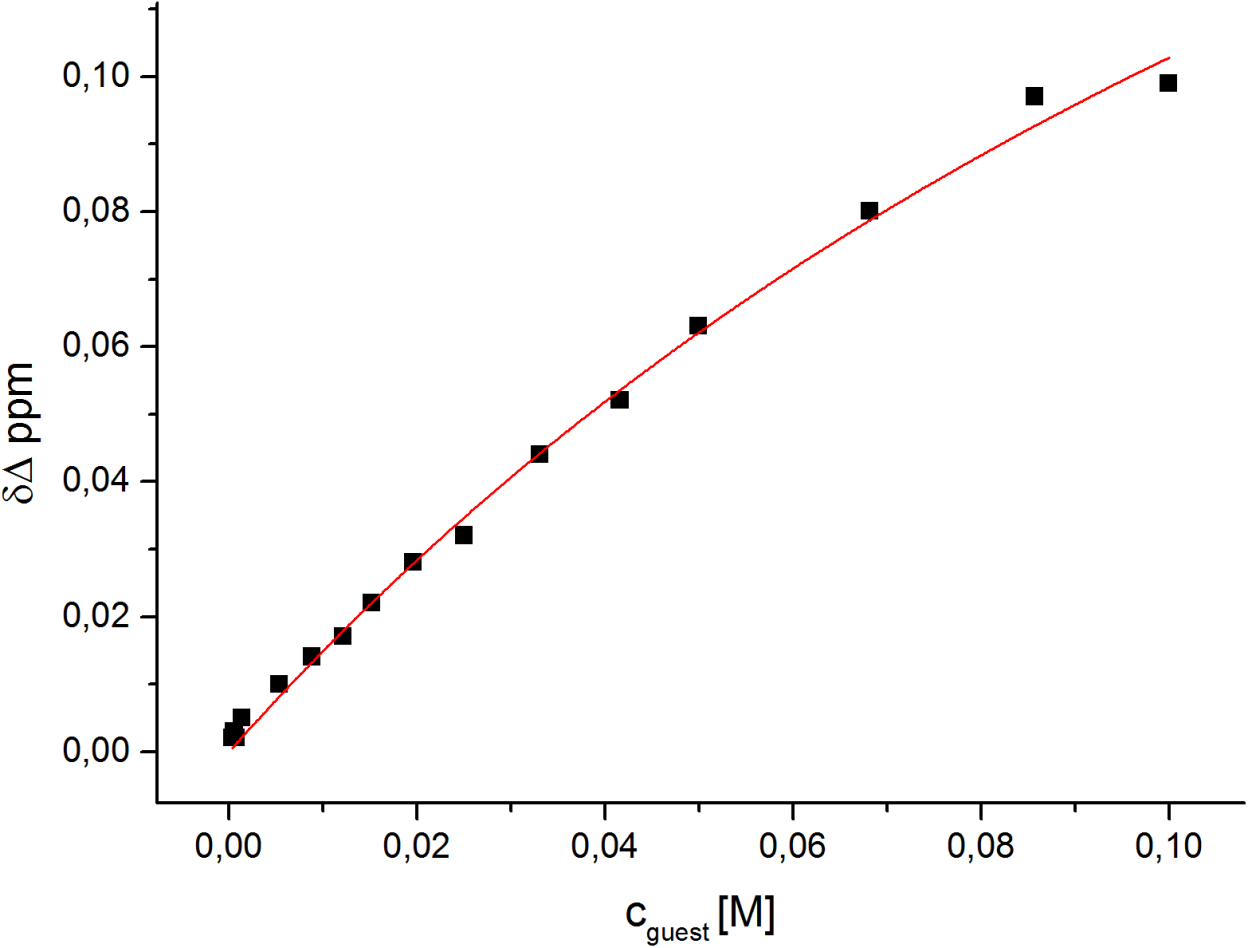
The best fit obtained for the ^1^H-NMR-titration of sodium 2-naphthoate into the PyrG4 dendrimer in aqueous solution. The calculated binding constant corresponds to K_*a*_ = 5.35±0.7 M^-1^ for *n* = 2 carboxylates.

However, the fitting algorithm could successfully be employed for the 2-naphthoate. The obtained association constant K_*a*_ = 5.35±0.7 M^-1^ (for *n* = 2 carboxylate molecules) is approximately in the same order of magnitude as the one determined by the ITC experiment. The best fit for the titration series is shown in Fig 6. The difference between the determined binding constant by ITC and NMR is most likely due to the assumptions made in the NMR model, e.g. equal contribution for first and second bound guest. In comparison, the ITC measures the actual heat output of the complexation, which include all contributions, e.g. conformational changes in the dendrimer structure, replacement of water with guest molecules etc. In contrast the NMR model correlates the chemical shift to a ratio between bound and unbound guest molecules, it neglects to take into account any structural changes incurred by the dendrimer during the binding process. Both in the ITC and the NMR study, the 2-naphthoate was found to exhibit weak binding to the G4 1-(4-carbomethoxy) pyrrolidone dendrimer.

## Conclusion

The G4 1-(4-carbomethoxy) pyrrolidone terminated PAMAM-dendrimer was found to form dendrimer-carboxylate complexes with both of the model compounds, sodium 2-naphthoate and sodium 3-hydroxy-2-naphthoate, in water. This was elucidated by NMR- and ITC-experiments. Complexation of the guest was found to occur in the interior of the dendrimer, possibly attributed to favorable lipophilic interactions. A difference in binding stoichiometry and binding strength was found for the two different model compounds. Both carboxylates had a primary stoichiometry of two guest molecules per dendrimer, presumably in close proximity to the dendrimer core. The 3-hydroxy-2-naphthoate derivative was found to associate more tightly with this binding site than the 2-naphthoate compound. The conducted ITC-experiments suggest an additional weak binding of app. 40 units of 3-hydroxy-2-naphthoate molecules to the interior branching of the G4 1-(4-carbomethoxy) pyrrolidone dendrimer.

These findings illustrate the potential of the G4 1-(4-carbomethoxy) pyrrolidone dendrimer to complex carboxylate guests and act as a possible nano-carrier of such molecules. Future studies on the potential of this dendrimer family as hosts for biologically active carboxylates, e.g. antibiotic and non-steroidal anti-inflammatory drugs, are currently in progress. This future work will also focus on the role of the cationand whether it too is complexed within the dendrimer cavities. Finally, the release of the bound carboxylate guests is also being investigated. The 1-(4-carbomethoxy) pyrrolidone surface also has synthetic “handles”, which can be utilized to link targeting units to the dendrimer.

## Supporting Information

S1 Fig The synthesis of a G4 1-(4-carbomethoxy-pyrrolidone) terminated PAMAM-dendrimer with 64 surface groups. (TIFF)

S2 Fig The ^1^H-NMR Assignment of the G4 1-(4-carbomethoxy-pyrrolidone) terminated PAMAM-dendrimer with 64 surface groups. (TIFF)

S3 Fig The ^13^C-NMR Assignment of the G4 1-(4-carbomethoxy-pyrrolidone) terminated PAMAM-dendrimer with 64 surface groups. (TIFF)

S4 Fig Stacked ^1^H-NMR-spectra showing the spectral change upon titration of the G4 4-carbomethoxy pyrrolidone PAMAM-dendrimer with sodium 3-hydroxy-2-naphthoate in D_2_O. (TIFF)

S5 Fig Stacked ^1^H-NMR-spectra showing the spectral change upon titration of the G4 4-carbomethoxy pyrrolidone PAMAM-dendrimer with sodium 2-naphthoate in D_2_O. (TIF)

S6 Fig The obtained Job plots for complex formation of sodium 3-hydroxy-2-naphthoate (left) and sodium 2-naphthoate (right) with the G4 1-(4-carbomethoxy) pyrrolidone dendrimer in water (D_2_O). (TIF)

S7 Fig 2D-NOE-spectrum showing correlation between sodium 3-hydroxy-2-naphthoate and the G4 1-(4-carbomethoxypyrrolidone) PAMAM-dendrimer. (TIF)

S8 Fig Graphic illustration picturing the assumed binding of the two units of 3-hydroxy-2-naphthoate within the dendrimer cavity in close proximity to the aliphatic butyl core. (TIF)

S9 Fig 2D-NOE-spectrum showing correlation between sodium 3-hydroxy-2-naphthoate and the G4 1-(4-carbomethoxypyrrolidone) PAMAM-dendrimer. (TIF)

S10 Fig ITC-heat signals for titration of sodium 3-hydroxy-2-naphthoate into 0.1 mM G4 4-carbomethoxy pyrrolidone terminated PAMAM-dendrimer. (TIF)

S11 Fig ITC-heat spectrum for titration of sodium 2-naphthoate into 0.1 mM G4 4-carbomethoxy pyrrolidone terminated PAMAM-dendrimer. (TIF)

S12 Fig ITC-heat spectrum for blank titration of sodium 2-naphthoate into MQ water. (TIF)

S13 Fig ITC-heat spectrum for blank titration of sodium 3-hydroxy-2-naphthoate into MQ water. (TIF)

S14 Fig ITC-heat spectrum for blank titration of water into 0.1 mM G4 4-carbomethoxy pyrrolidone terminated PAMAM-dendrimer. (TIF)

S1 Text Experimental details concerning dendrimer synthesis and characterization as well as further ITC information. (DOCX)
