# Supplementary figures and images for: Guest-Host Chemistry with Dendrimers—Binding of Carboxylates in Aqueous Solution
Source: PLoS One. 2015 Oct 8;10(10):e0138706. doi: 10.1371/journal.pone.0138706 (PMC4598172; doi:10.1371/journal.pone.0138706)

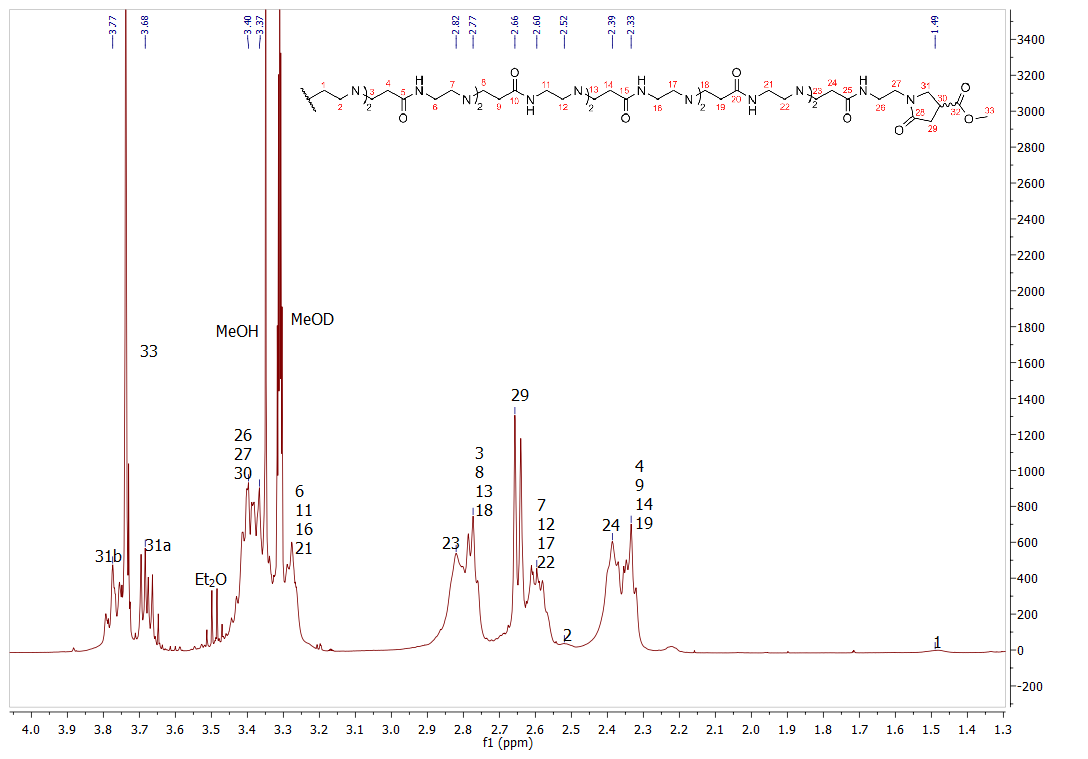

Supplement: S2 Fig — (TIFF) [file pone.0138706.s002.tiff]

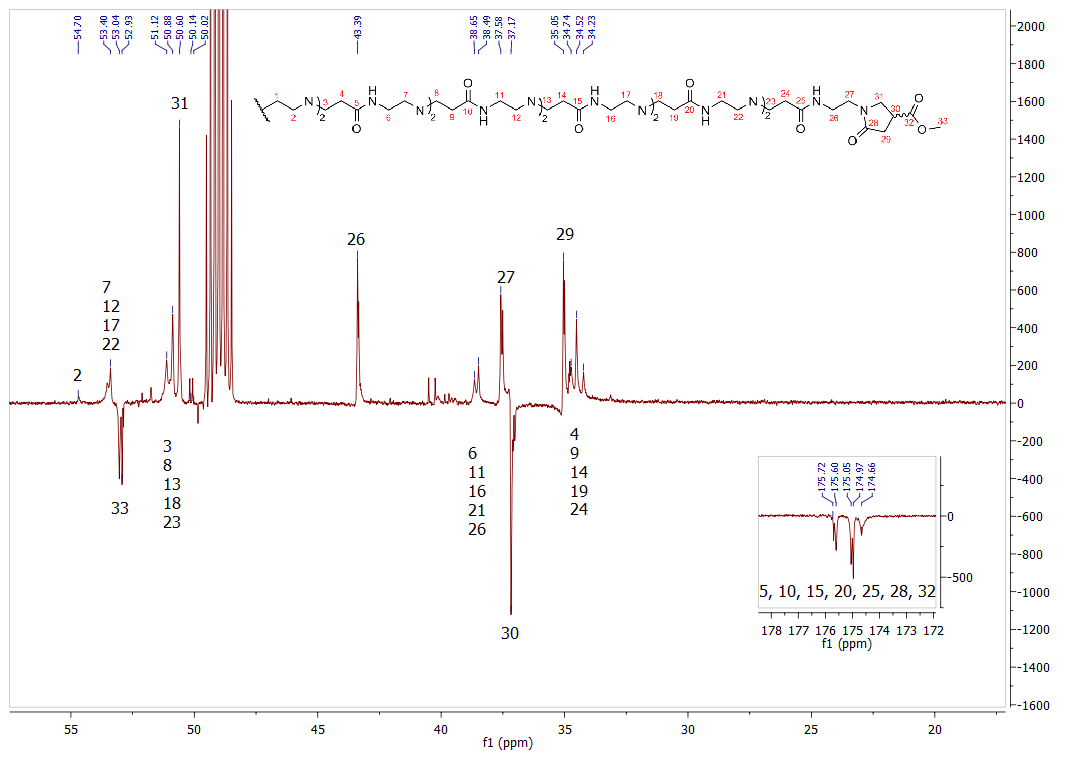

Supplement: S3 Fig — (TIFF) [file pone.0138706.s003.tiff]

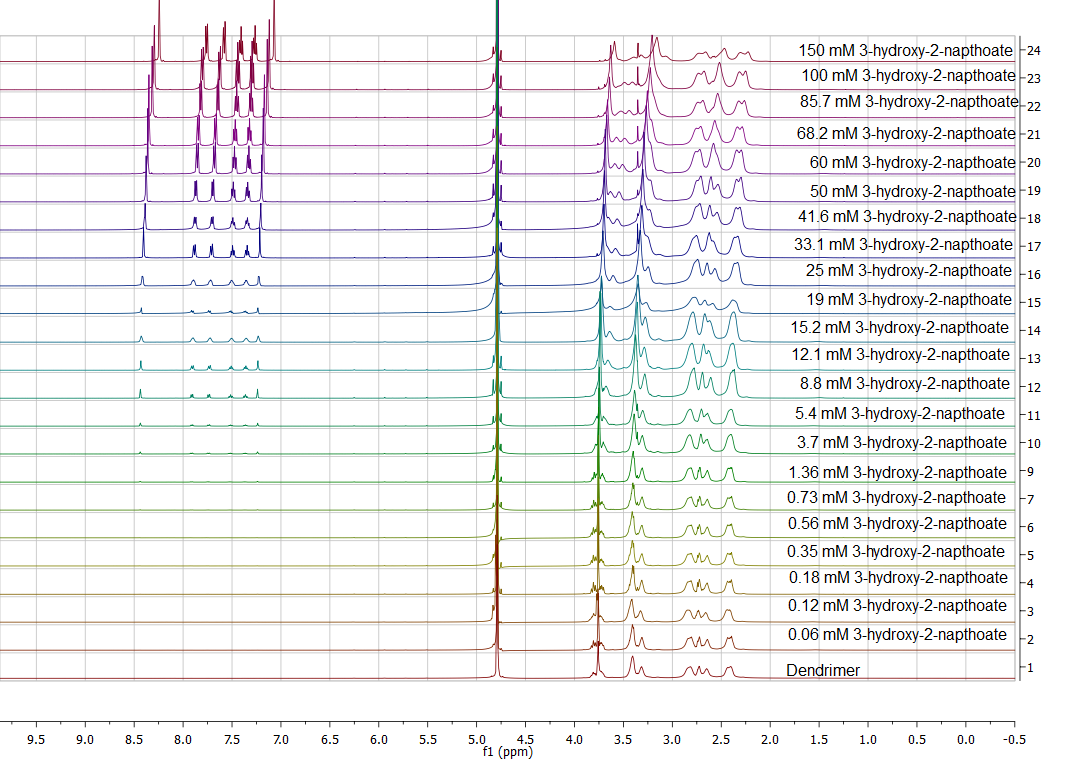

Supplement: S4 Fig — (TIFF) [file pone.0138706.s004.tiff]

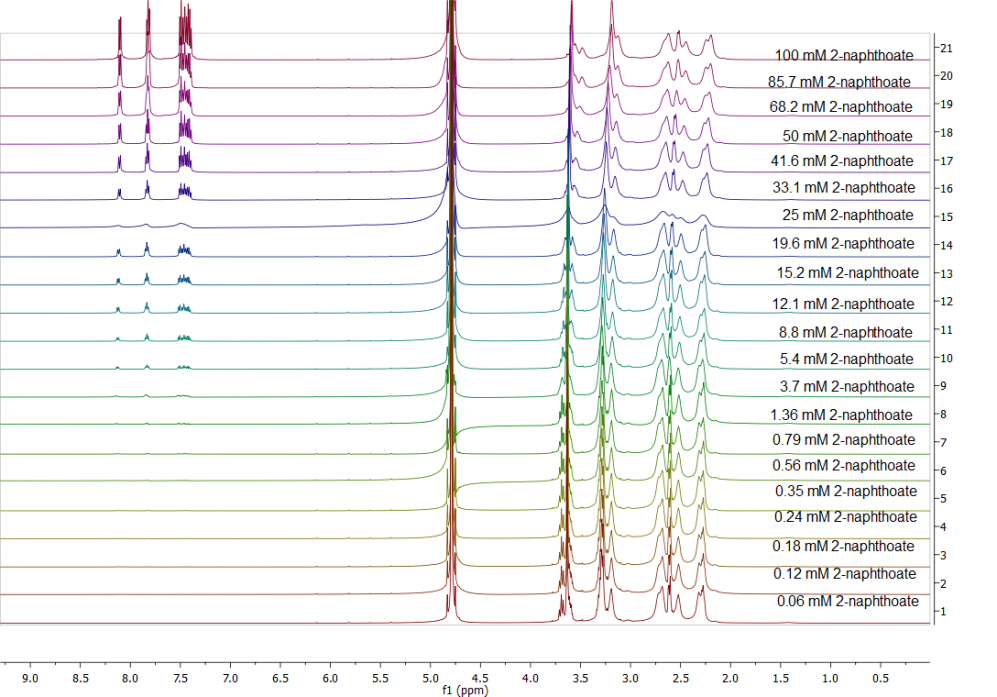

Supplement: S5 Fig — (TIF) [file pone.0138706.s005.tif]

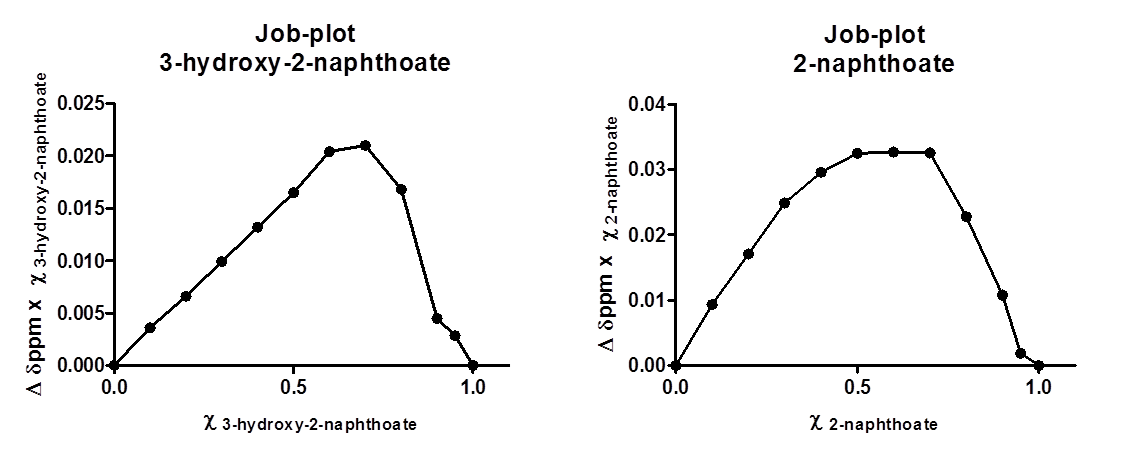

Supplement: S6 Fig — (TIF) [file pone.0138706.s006.tif]

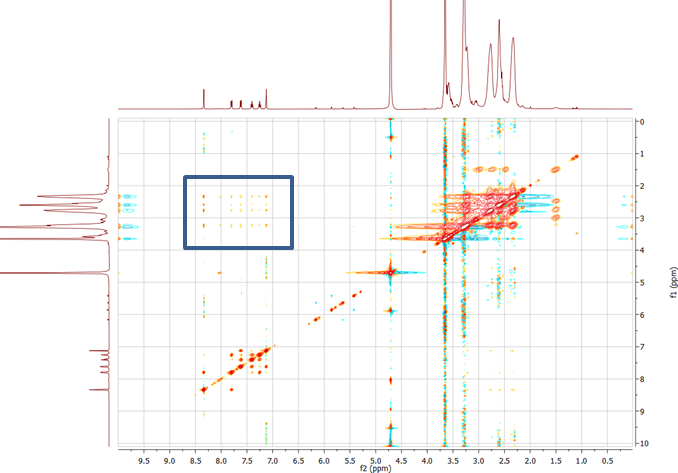

Supplement: S7 Fig — (TIF) [file pone.0138706.s007.tif]

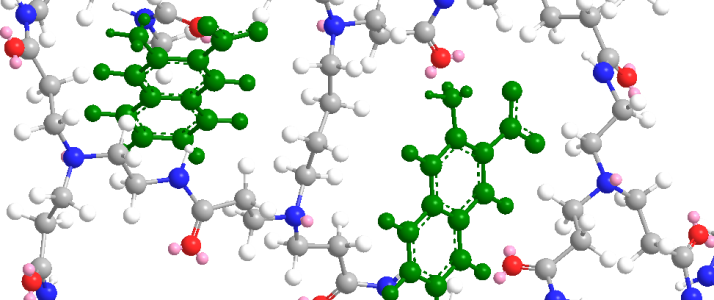

Supplement: S8 Fig — (TIF) [file pone.0138706.s008.tif]

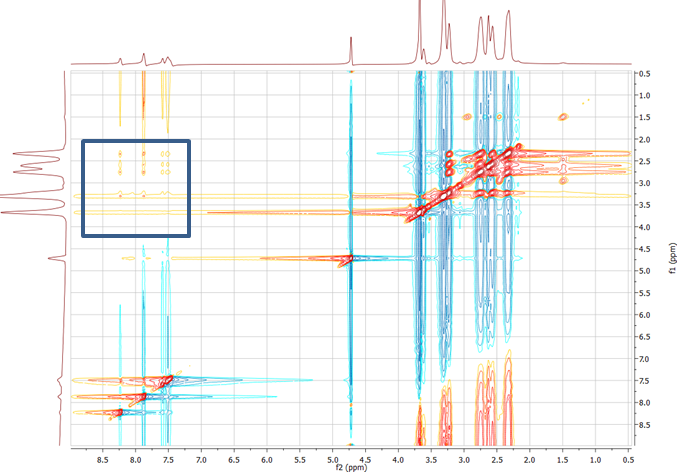

Supplement: S9 Fig — (TIF) [file pone.0138706.s009.tif]

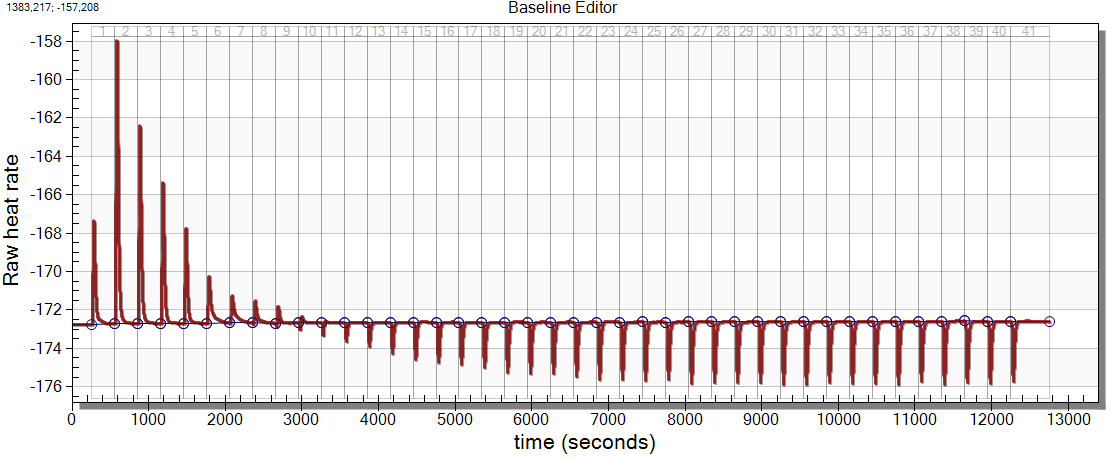

Supplement: S10 Fig — (TIF) [file pone.0138706.s010.tif]

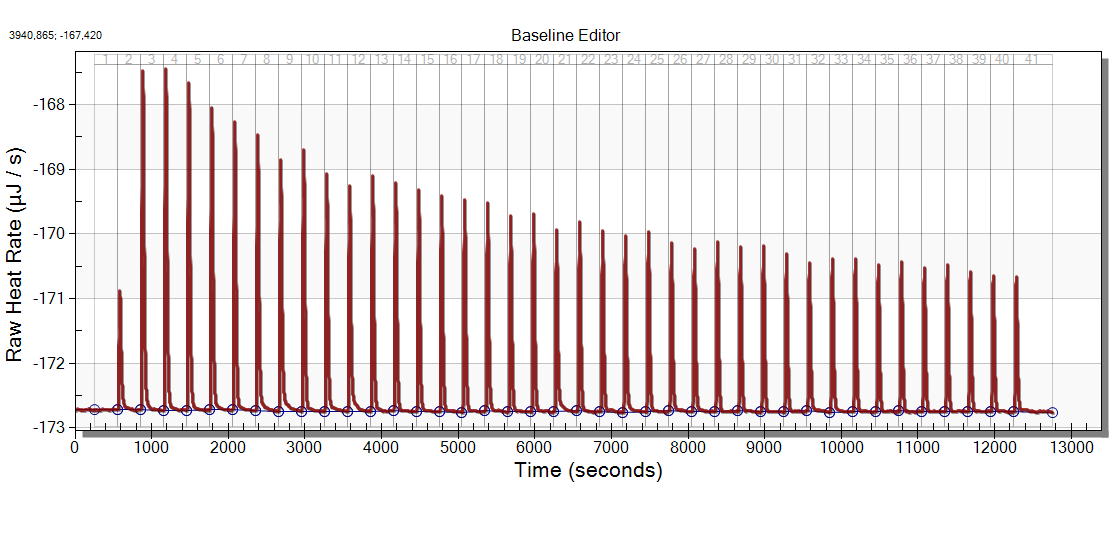

Supplement: S11 Fig — (TIF) [file pone.0138706.s011.tif]

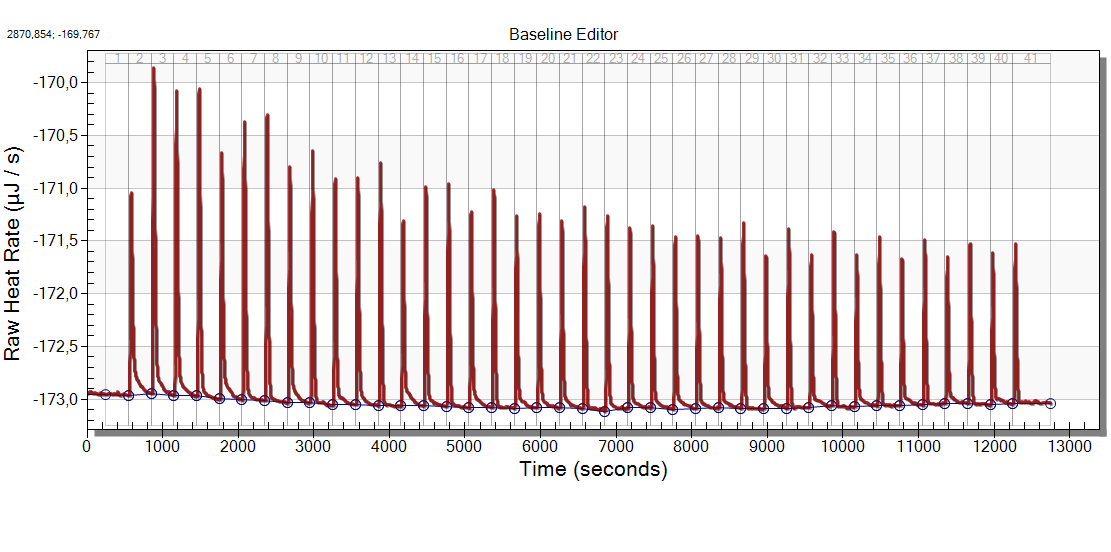

Supplement: S12 Fig — (TIF) [file pone.0138706.s012.tif]

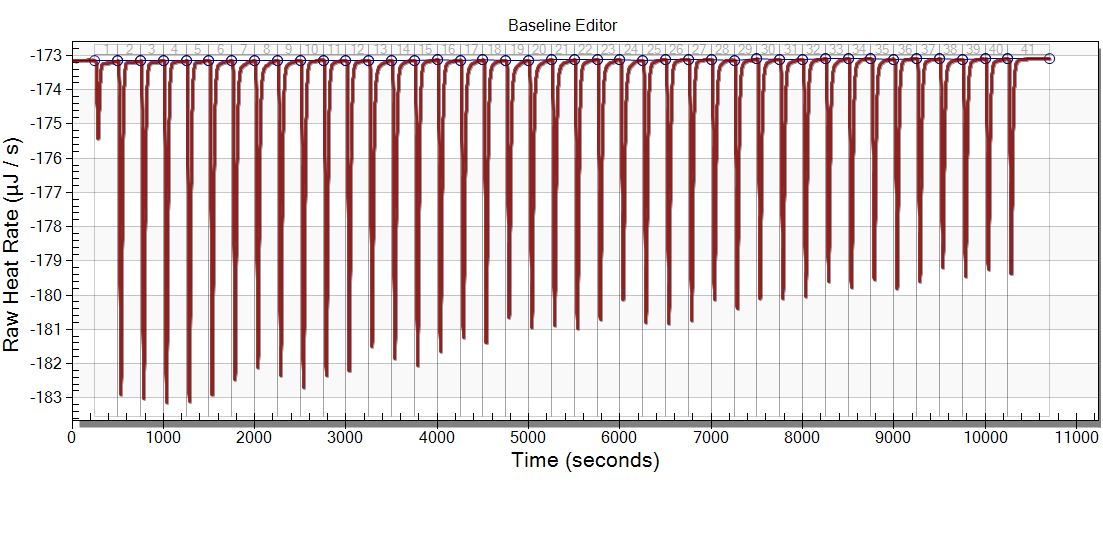

Supplement: S13 Fig — (TIF) [file pone.0138706.s013.tif]

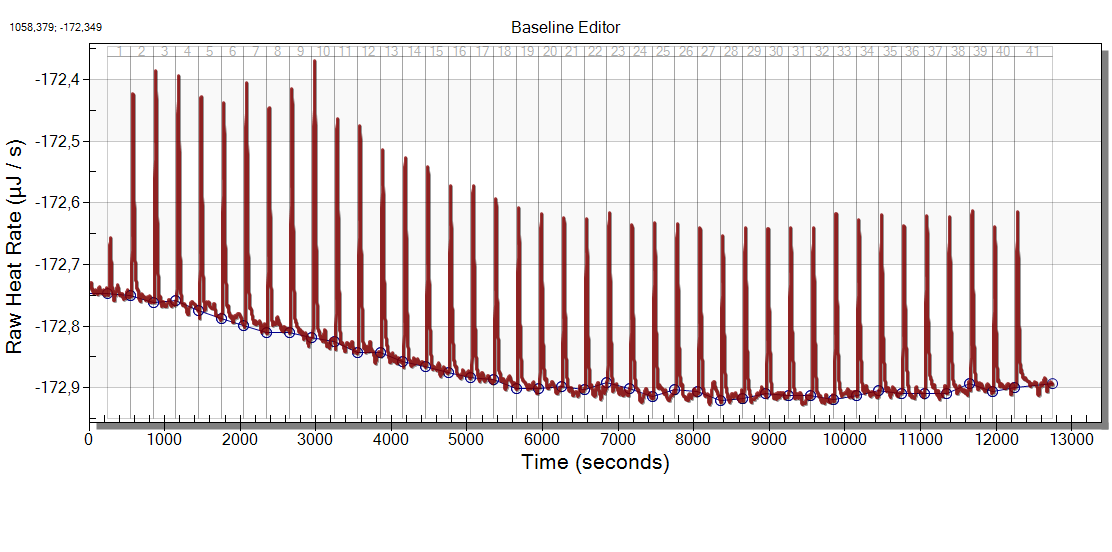

Supplement: S14 Fig — (TIF) [file pone.0138706.s014.tif]
