# Supplementary material for: Guest-Host Chemistry with Dendrimers—Binding of Carboxylates in Aqueous Solution
Source: PLoS One. 2015 Oct 8;10(10):e0138706. doi: 10.1371/journal.pone.0138706 (PMC4598172; doi:10.1371/journal.pone.0138706)
Supplement: S1 Text — (DOCX) [file pone.0138706.s015.docx]

**Supporting Information**

**Text S1. Experimental details concerning dendrimer synthesis and characterization as well as further ITC information.**

Preparation and characterization of the 1-(4-carbomethoxy) pyrrolidone dendrimer

Amino-terminated PAMAM-dendrimers were synthesized according to literature methods [24], ^1^H- and ^13^C-NMR data was recorded and compared to the published analytical data. Surface functionalization the 4-carbomethoxy pyrrolidone was achieved by reaction with dimethyl itaconate in methanol [6, 25]. A 15 w/w% solution of G4-PAMAM dendrimer ( 1.8 g, 0.126 mmol) was added slowly to a 70 w/w% solution of dimethyl itaconate in methanol (1.34 g, 8.5 mmol, 1.05 equivalents per amino functionality), while cooling with a water/ice bath. Upon completion of the addition the reaction mixture was left in the cooling bath to slowly heat to ambient temperature. The reaction was stirred until the Kaiser-test (ninhydrin in ethanol) was negative, usually 3-4 days. The functionalized dendrimer was purified by dialysis against first methanol and then water. The aqueous phase was freeze-dried to give the pure pyrrolidone-terminated PAMAM-dendrimers (2.6 g, 0.118 mmol, 91 %) as white, very hygroscopic solids. For analytical characterization see Figure S2 for the ^1^H-NMR assignment and Figure S3 for the ^13^C-NMR assignment.

Isothermal Calorimetry

Isothermal titration calorimetry allows studying thermodynamic properties of macromolecular systems with guest molecules by measuring the heat release after the addition of a defined amount of guest molecule to a host molecule. Measuring multiple of these heat signals in a titration series gives inside in the binding affinity of the guest to the host molecule. For a detailed understanding of the technique and the thermodynamic principles/equations behind it, we would like to refer to reference [31] by Freire et. al. which is an excellent theoretical background publication about the technique. For an experimental best practice guideline, we followed and refer to the publication by Freyer and Lewis [26, 31].

For each individual injection a heat signal is obtained, this signal is a consequence of (equations were derived in literature reference [31]):

$Q=V\Delta H\sum\Delta\left[ Guest \right]=V\Delta H[Guest]$ (1)

$Q=V[Dendrimer]\frac{n\Delta HK[Guest]}{1+K[Guest]}$ (2)

The cumulative heat (Q) in equation 1 dependents on the reaction volume (V), the binding enthalpy (ΔH) and the concentration of bound guest ([Guest]). This can be rewritten, taking into account that the concentration of bond guest is a function of the dendrimer concentration ([Dendrimer]) and the binding constant (K) and the number of independent binding sites per dendrimer (n).

For the dendrimer system with two sets of independent binding sites the equation needs to be extended for the second K value (K_2_), enthalpy (ΔH_2_) and number of binding sites (n_2_), which makes the computational fitting (*NanoAnalzse* software) less precious, since multiple parameters needs to be fitted with the same amount of information (heat signal Q), thus the error values for each parameter increases.

$Q=Q_{1}+Q_{2}=V(\Delta H_{1}\left[ Guest,Binding Site 1 \right]+\Delta H_{2}\left[ Guest,Binding Site 2 \right])$ (3)

$Q=V[Dendrimer](\frac{n_{1}\Delta H_{1}K_{1}\left[ Guest, Binding Site 1 \right]}{1+K_{1}\left[ Guest, Binding Site 1 \right]}+\frac{n_{2}\Delta H_{2}K_{2}\left[ Guest, Binding Site 2 \right]}{1+K_{2}\left[ Guest, Binding Site 2 \right]}$) (4)

**S1 Fig. The synthesis of a G4 1-(4-carbomethoxy-pyrrolidone) terminated PAMAM-dendrimer with 64 surface groups.**

**S2 Fig. The ^1^H-NMR Assignment of the G4 1-(4-carbomethoxy-pyrrolidone) terminated PAMAM-dendrimer with 64 surface groups.**

**S3 Fig. The ^13^C-NMR Assignment of the G4 1-(4-carbomethoxy-pyrrolidone) terminated PAMAM-dendrimer with 64 surface groups.**

**S4 Fig. Stacked ^1^H-NMR-spectra showing the spectral change upon titration of the G4 4-carbomethoxy pyrrolidone PAMAM-dendrimer with sodium 3-hydroxy-2-naphthoate in D_2_O.**

**S5 Fig. Stacked ^1^H-NMR-spectra showing the spectral change upon titration of the G4 4-carbomethoxy pyrrolidone PAMAM-dendrimer with sodium 2-naphthoate in D_2_O.**

**S6 Fig. The obtained Job plots for complex formation of sodium 3-hydroxy-2-naphthoate (left) and sodium 2-naphthoate (right) with the G4 1-(4-carbomethoxy) pyrrolidone dendrimer in water (D_2_O).**

**S7 Fig.** **2D-NOE-spectrum showing correlation between sodium 3-hydroxy-2-naphthoate and the G4 1-(4-carbomethoxypyrrolidone) PAMAM-dendrimer.**

**S8 Fig. Graphic illustration picturing the assumed binding of the two units of 3-hydroxy-2-naphthoate within the dendrimer cavity in close proximity to the aliphatic butyl core.**

**S9 Fig. 2D-NOE-spectrum showing correlation between sodium 3-hydroxy-2-naphthoate and the G4 1-(4-carbomethoxypyrrolidone) PAMAM-dendrimer.**

**S10 Fig.**  **ITC-heat signals for titration of sodium 3-hydroxy-2-naphthoate into 0.1 mM G4 4-carbomethoxy pyrrolidone terminated PAMAM-dendrimer.**

**S11 Fig. ITC-heat spectrum for titration of sodium 2-naphthoate into 0.1 mM G4 4-carbomethoxy pyrrolidone terminated PAMAM-dendrimer.**

**S12 Fig.**  **ITC-heat spectrum for blank titration of sodium 2-naphthoate into MQ water.**

**S13 Fig.** **ITC-heat spectrum for blank titration of sodium 3-hydroxy-2-naphthoate into MQ water.**

**S14 Fig.**  **ITC-heat spectrum for blank titration of water into 0.1 mM G4 4-carbomethoxy pyrrolidone terminated PAMAM-dendrimer.**
